# Supplementary material for: Genetic strategies for sex-biased persistence of gut microbes across human life
Source: Nat Commun. 2023 Jul 14;14:4220. doi: 10.1038/s41467-023-39931-2 (PMC10349097; doi:10.1038/s41467-023-39931-2)
Supplement: Supplementary file 1 — Supplementary Information [file 41467_2023_39931_MOESM1_ESM.pdf]

Genetic strategies for sex-biased persistence of gut microbes across human life

Running title: Host's gender-specific gut microbiome engraftment

Key words: infant, microbiome, strain-level, persistence, gender, Bifidobacterium

Chiara Tarracchini[1], Giulia Alessandri[1], Federico Fontana[1,2], Sonia Mirjam Rizzo[1], Gabriele Andrea Lugli[1], Massimiliano Giovanni Bianchi[3], Leonardo Mancabelli[1], Giulia Longhi[1,2], Chiara Argentini[1], Laura Maria Vergna[1], Rosaria Anzalone[2], Alice Viappiani[2], Francesca Turroni[1,4], Giuseppe Taurino[3], Martina Chiu[3], Silvia Arboleya[5], Miguel Gueimonde[5], Ovidio Bussolati[3,4], Douwe van Sinderen[6], Christian Milani[1,4*], Marco Ventura[1,4*].

[*] These authors contributed equally

Laboratory of Probiogenomics, Department of Chemistry, Life Sciences, and Environmental Sustainability, University of Parma, Parma, Italy[1]; GenProbio srl, Parma, Italy[2]; Laboratory of General Pathology, Department of Medicine and Surgery, University of Parma, Parma, Italy[3]; Microbiome Research Hub, University of Parma, Parma, Italy[4]; Department of Microbiology and Biochemistry of Dairy Products, Instituto de Productos Lácteos de Asturias, CSIC, 33300 Villaviciosa, Spain[5]; APC Microbiome Institute and School of Microbiology, Bioscience Institute, National University of Ireland, T12YT20, Cork, Ireland[6]

[*]Correspondence

Mailing address for Marco Ventura, Laboratory of Probiogenomics, Department of Chemistry, Life Sciences, and Environmental Sustainability, University of Parma, Parco Area delle Scienze 11a, 43124 Parma, Italy. Phone: ++39-521-905666. Fax: ++39-521-905604. E-mail: marco.ventura@unipr.it

Mailing address for Christian Milani, Laboratory of Probiogenomics, Department of Chemistry, Life Sciences, and Environmental Sustainability, University of Parma, Parco Area delle Scienze 11a, 43124 Parma, Italy. Phone: ++39-0521-904785. E-mail: christian.milani@unipr.it

Supplementary text

Microbial taxonomic profiling of the infant gut microbiota. Taxonomic dissection of the infant gut microbiomes showed that the bacterial species richness ranged from an average of 12.3 species, observed over the first months of life, to an average of 28.5 detected at 24 months of age, corroborating the well-known progressive increase in bacterial species richness proportional to the infant age (Supplementary Data 2) (1,2). Furthermore, analysis of the overall compositional profiles of the gut microbial communities represented through Principal Coordinate Analysis (PCoA) revealed differences in taxonomic compositions that could be linked with age, with the 24-months old infants and younger subjects encompassing distinct clusters in the PCoA (Figure S2). Taken together, these data showed that the largest shifts in structure and composition of the infant gut microbiome happen between 6 and 12 months, likely triggered by dietary changes occurring during this time, thus supporting the notion that the weaning process has a marked impact on shaping the development of the infant gut microbiota (3,4).

Consistent with previous scientific literature focusing on dissecting the infant gut's recurring microbial patterns (i.e., community state types), although there is high inter-individual variability in the gut microbiome through infancy, higher relative abundances of the above-mentioned bifidobacterial species were found in 1–6-month-old breastfed infant feces compared to those from age-matched formula-fed newborns (Supplementary Data 2) (5,6). Furthermore, members of the *Bifidobacterium* genus were detected up to 24 months after birth, highlighting their ability to establish long-term colonization in the infant gut (Figure S3, Figure S4, Supplementary Data 2) (4). Specifically, members of *B. longum* species have been detected in infant feces until the age of one, demonstrating their persistence in the infant gut beyond weaning. In particular, newborns who received breast milk between one and six months post-birth showed an average persistence of *B. longum* species of 100 % at 12 months (with an

average abundance of 4.78 %), which was higher compared to the 12-months *B. longum* persistence among formula-fed infant population (66.6 % with an average abundance of 1.81 %) (Figure S4, Supplementary Data 2). However, as it has been widely reported, the relative average abundances of the above-mentioned bifidobacterial species as well as *E. coli* continue to decrease with aging (Kruskal-Wallis test, p-value < 0.01), while the gut microbiome of 24-months old infants get more complex by enriching with adult-like bacterial species, such as *Eubacterium rectale*, *Faecalibacterium prausnitzii*, *Ruthenibacterium lactatiformans*, *Akkermansia muciniphila*, and members of the *Bacteroides* genus, such as *Bacteroides uniformis* (Figure S3, Supplementary Data 2) (7,8).

As mentioned, WMS data could be exploited to assemble draft genomes of uncultivated bacteria from biological samples harboring complex microbial communities (9). In this context, the bacterial reads that passed quality filtering and human genome mapping were used for *de novo* metagenomics assembly and taxonomic classification of contigs using METAnnotatorX2, as previously applied (10,11). In detail, considering the 11 above-mentioned main infant gut-associated bacterial species, we efficiently assembled 64 bacterial genomes from the available metagenomic data, obtaining a minimum average reads coverage of 12X and a total genome size compatible with what is reported in literature (Supplementary Data 3). The pool of draft genomes was employed to generate 11 species-specific databases that were enriched with the available high-quality genomes (complete and draft) in public repositories.

Strain diversity and persistence of the most post-weaning abundant species. To gain more insights into the dynamics of the infant gut microbiome, we considered the five above-listed bacterial species mainly associated with the gut of weaned infants (12-24 months old), i.e., *Eubacterium rectale*, *Faecalibacterium prausnitzii*, *Ruthenibacterium lactatiformans*, *Akkermansia muciniphila*, and *Bacteroides uniformis*, with an average abundance ranging from

8.22 % for *E. rectale* to 1 % for *R. lactatiformans* (Supplementary Data 2). Moreover, these selected species were detected both at 12 and 24 months of age, with a prevalence ranging from 27 % for *A. muciniphila* to 45 % for *E. rectale* (Supplementary Data 2).

Comparison of the gut strain community structure between 12 and 24 months provided intriguing results, as it allows observing changes among samples across a one-year time span. For each infant, these evaluations were achievable for up to four of the five above-described species since not all the examined species were simultaneously present at 12 and 24 months (Figure S3, Figure S4, Supplementary Data 2). However, our data show that up to 100 % (average of 81 %) of the strains dominating at 12 months were replaced by new dominant strains at 24 months (Figure S3, Figure S4).

For example, microbial consortia belonging to *F. prausnitzii* in Infant_3 at 12 months encompassed three strains, none clearly dominating. Notably, one of these strains was replaced with a new genetic variant at the 24-month time point while another became dominant. Moreover, in formula-fed Infant_5, Infant_6, and Infant_10, members of the *R. lactatiformans* species [a Ruminococcaceae member generally isolated from adult human feces (12)] were measurable at pre-weaning time-points in addition to 12-24 months of age, thus allowing an extensive tracking of strains over time (Figure S3, Figure S4). Accordingly, we observed that at 1-6 months, all three infants showed a single-strain *R. lactatiformans*, while at the 12-month time point, a new genetic variant appeared, becoming the dominant strain at 24 months of age (Figure S3, Figure S4). Remarkably, these data suggest how specific strains of pioneering species tend to persist through later stages of life, probably constituting a reservoir for a new generation, while strain populations of species typically associated with adulthood, acquired post-weaning due to a more variable solid diet, tend to be subjected to continual rearrangements with limited strain persistence.

The strain change-index was obtained for each infant, calculating the ratio between the number of species that undergoes strain-level qualitative or quantitative changes between two time points of interest and the number of species for which such inter-timing comparison was possible. Interestingly, the array of such values obtained among the weaned infant population (12-24 months old, average change-index = 0.83) was greater compared to those acquired from suckling infants (1-6-months old, average change-index = 0.61) (Mann–Whitney U, p-value < 0.05). These results suggest that rearrangements in terms of strain composition occur more frequently between 12 and 24 months-old infants rather than between pre- and post-weaning newborns. This is likely due to the presence of (bifido)bacterial species inhabiting the suckling infant' gut that, especially in those who received breastfeeding, establish a stable microbial consortium even across the weaning process. However, although several previous studies reported a species-level microbiome relatively unchanged between 12 and 24 months (13,14), these observations collectively suggest obvious changes in strain-level relative abundances and noticeable structure rearrangement of the infant gut-associated microbiome.

The Glycosyl hydrolase arsenal did not support the superior persistence of (certain) bifidobacterial strains. Although a high persistence over the first two years of life was observed among the early engrafted bifidobacterial strains, the appearance of different conspecific genetic variants that failed to colonize the infant gut permanently (or accomplished it in the more advanced phases of the weaning process) was frequently noted (Figure S5). Therefore, we attempted to functionally explain the higher persistence of certain bifidobacterial strains through glycobiome analysis since glycosyl hydrolases are renowned for their involvement in host-microbe interaction. However, glycobiome inspection of strains metagenomically assembled from infants (eleven persistent and six transient bifidobacterial genomes belonging to *B. longum*, *B. bifidum*, *B. pseudocatenulatum*, and *B. breve* species)

appeared not to provide relevant genetic support for explaining the increased strain stability phenotype. This outcome could be due to the limited number of considered strains as well as their belonging to different species, which altogether make obtaining powered across-species comparisons challenging.

Insights into the genetic repertoire associated with microbial persistence. Comparative genome analyses between persistent species (*B. bifidum* and *B. longum*) and the microbial species showing poor persistence capability revealed the presence of 14 protein families named PDCs (Persistence Determinant COGs). Among these, a gene containing a structural domain shared with a serine/threonine-protein kinase (STPK) (COG_1326) deserves our interest due to its possible implication in enhancing persistence (Figure 1b, Supplementary Data 10) (15,16). In addition, within the gene pool characteristic of these latter species, we found an FHA-domain containing protein (forkhead-associated domain, the substrate of STPK) (COG_1300) (Figure 2b, Supplementary Data 10), corroborating the possible role of protein phosphorylation in the microbial species-specific strategies for achieving persistence (15).
Another intriguing PDC is COG_1307, encoding a 4'-phosphopantetheinyl transferase superfamily protein. These proteins are involved in post-translational modifications required for carrier proteins maturation as well as activation of the acyl-carrier protein domain(s) of enzymes such as polyketide synthases, fatty acid synthases, and non-ribosomal peptide synthetases (38). The latter produce unusual polyketides, atypical fatty acids, peculiar antibiotics such as surfactins, and N-Acyl homoserine lactones which mediate quorum sensing (39). Furthermore, the PDC COG_1315 encoding the protein domain “Virulence factor BrkB”, in *Bordetella pertussis* has been demonstrated to be essential for resistance to complement-dependent killing by human serum (40). Thus, suggesting a putative role in the interaction with the host’s immune system. Remarkably, in 100 % of *B. bifidum* genomes and 79 % of those

belonging to *B. longum* we identified a PDC known to encode for an extracellular membrane-anchored glycosyl hydrolase family 101 (GH101) (COG_1440). This enzyme, acting on mucin glycan core structures, can support the long-lasting colonization of mucin-degrading bacteria in the infant gut by providing an endogenous source of nutrients in the absence of dietary glycans (41).

Similarly worthy of notice is a PDC (COG_2243) found in each screened *B. bifidum* genome, and 38 % of those belonging to *B. longum* species (Figure 1b; Supplementary Data 10). In *B. longum* subsp. *longum*, the crystal structure analyses of the catalytic domain have been reported in a recent study, leading to the establishment of the novel glycoside hydrolase family 136 (GH136) acting as an extracellular lacto-N-biosidase (42). In contrast, the corresponding encoded protein in *B. bifidum* species has not yet been characterized, although it shares about the 59 % of sequence similarity and the main protein domains with the GH136 gene of *B. longum* (38,39) (Figure S6a, b). Regarding the remaining PDCs, six PCDs have been predicted to exploit a range of functions for which direct correlations with persistence are currently unknown, such as two acetyltransferases, two aminotransferases, a metalloprotease, and a transporter with unidentified specificity. Moreover, no indications regarding biological functions could be retrieved for PDCs COG_1442 and COG_1601, representing intriguing targets for further investigations.

A transcriptomic survey on *B. bifidum* PRL2010 leads to the identification of a link between host glycans metabolism and microbial persistence. Considering the renowned importance of host glycans in mediating host-microbe interactions (51), we evaluated the possible involvement of the 14 PDCs in the host glycan metabolisms. Interestingly, transcriptomic data based on *B. bifidum* PRL2010 grown on different carbon sources highlighted that the gene expression of the accessory GH136 (COG_2243) and the ubiquitous

GH101 (COG_1440), along with another PDC encoding an ABC transporter with unidentified specificity were up-regulated when 2'-fucosyllactose (2'-FL), 3-fucosyllactose (3-FL), 3′-sialyllactose (3'-SL), 6′-sialyllactose (6'-SL), and mucin were used as the sole carbon source rather than glucose (MRS-based medium) (Supplementary Data 10). These transcriptomics data indicated that GH136, GH101, and the PDC encoding an ABC transporter with unknown predicted specificity, as defined based on the Transporter Classification Database (TCdb) (52), may be implicated in the uptake and metabolism of specific host glycans and their derivates (Supplementary Data 11).

Phylogenetic distribution of PDCs. Phylogenomic analyses based on all the publicly available *B. bifidum* genomes revealed that the 14 PDCs are unevenly distributed across the strains extracted from populations with respect to the evolutionary dynamics of this species, with 11 of the 14 PDCs appearing to be a part of the *B. bifidum* core genome (Figure S7). Interestingly, these data are compatible with the reported persistence of this species across the whole human lifespan.

Conversely, within *B. longum* species, clear correlations between subspecies phylogeny and specific PDCs were revealed (Figure S7). In particular, the ubiquity of the PDC GH136 among subspecies *infantis* members contrasts with its strain-specific presence within the subspecies *longum*. Nevertheless, *B. longum* subsp. *longum* possesses a genomic architecture enriched in six PDCs compared to *B. longum* subsp. *infantis*, including PDCs encoding for the SRKP and GH101. This result is consistent with previous observations that this latter subspecies, which preferentially consumes smaller HMO molecules (degree of polymerization <7) (47), tends to colonize the lactating infant microbiome and is then lost in later stages of life. In contrast, the subspecies *longum* appears to persist across the host lifespan, being widely distributed in both infants and adults (47).

PDCs encoding for a STPK-FHA system may participate in modulation of the molecular machinery responsible for regulated host glycans-mediated microbial persistence.

In addition to the PDCs encoding GH136, GH101, and the putatively correlated ABC transporter, whose link with host glycans metabolism was demonstrated through RNA-Seq based approaches, the predicted PDCs also encompass a serine/threonine-protein kinase (STPK) (COG_1326) and an FHA-domain containing protein (forkhead-associated domain) (COG_1300), which represent the putative substrate of STPK (Figure 1b, Figure 2b, Supplementary Data 10). As a result of combined RNA-Seq experiments of (bifido)bacteria and human intestinal cell lines, serine/threonine-protein kinases deserve interest due to their possible role in host-microbe two-way interaction. Indeed, according to previous studies, it is plausible to suppose that STPK of *Bifidobacterium* can be elements of signal transduction networks playing a role in modulating biological functions in microbial cells, including bacterial adhesion and colonization of host's specific niches and modulation of the host's immune system (50,51). Moreover, bacterial kinases are capable of inducing changes in the host genes expression, as well as directly (post-translationally) modifying the host-proteome (52). Remarkably, a previous comparative genomics analysis evidenced this STPK protein uniquely in *B. bifidum* and *B. longum* species and identified its possible role in the adaptive cellular response (53). These findings suggest a possible role of protein phosphorylation in microbial species-specific strategies for achieving persistence. Accordingly, the PDCs GH136, GH101, ABC transporter, STPK, and FHA-domain containing protein can constitute complex molecular machinery that deserves further investigation for its possible role in regulating host glycans-mediated bifidobacterial persistence.

METHODS

***B. longum* and *B. bifidum* core-genome-based phylogenomic tree.** A selection of publicly available high-quality assemblies of *B. longum* (n=453) and *B. bifidum* genomes (n=96) (complete and dr

aft with a number of contigs < 90) were subjected to PGAP analysis as described above. Accordingly, the sequences of protein families identified as constituents of the *B. longum* core genome were concatenated and subsequently aligned using MAFFT (62). The resulting phylogenomic tree was constructed using the neighbor-joining method in ClustalW v2.1 (63) and visualized through Figtree v1.4 software (http:/tree.bio.ed.ac.uk/software/figtree/).

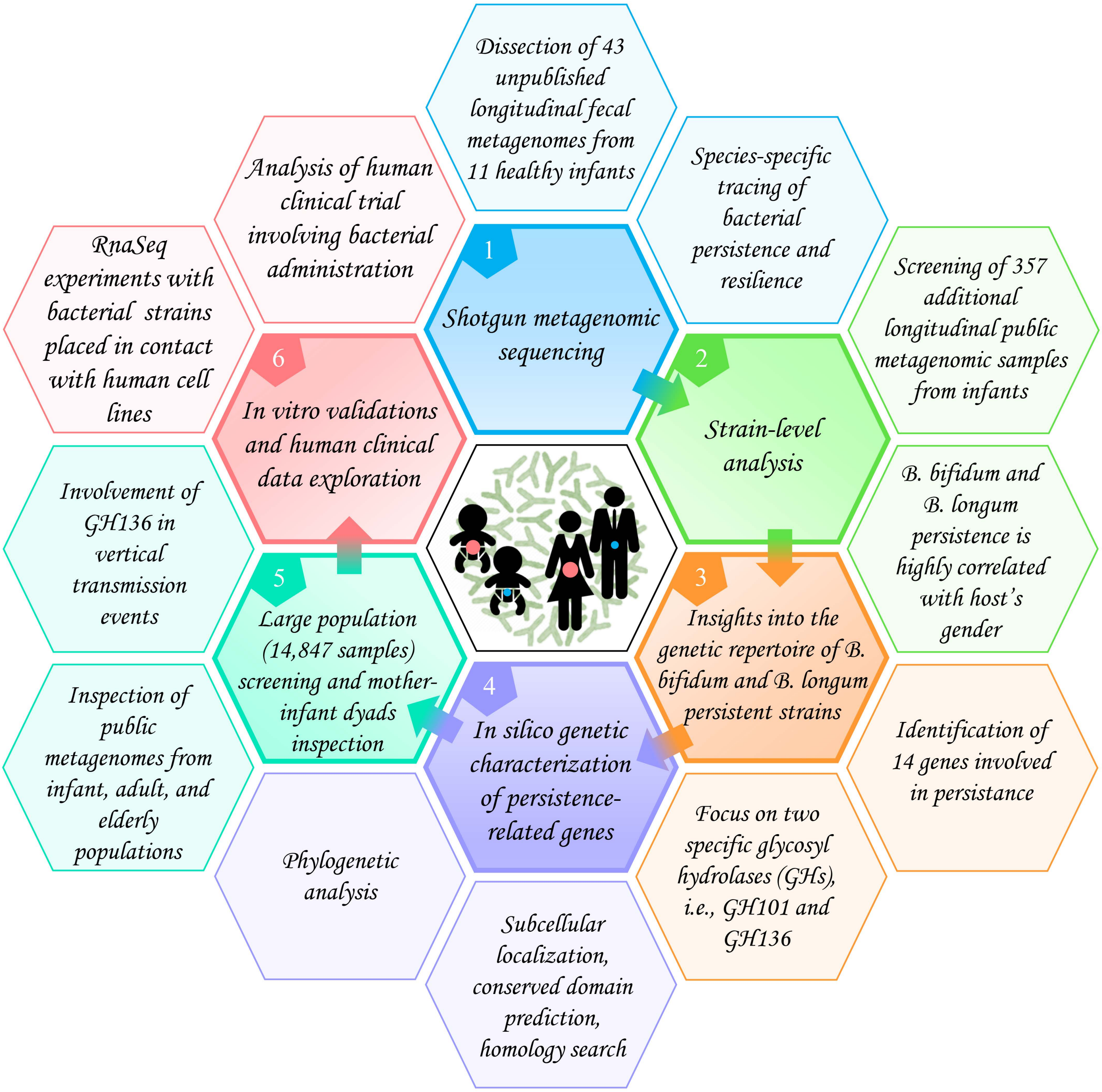

Figure S1. Schematic representation of the workflow employed in this study. Workflow steps were highlighted in different colors. The internally numbered hexagons represent the crucial study phases, while the external ones report additional details.

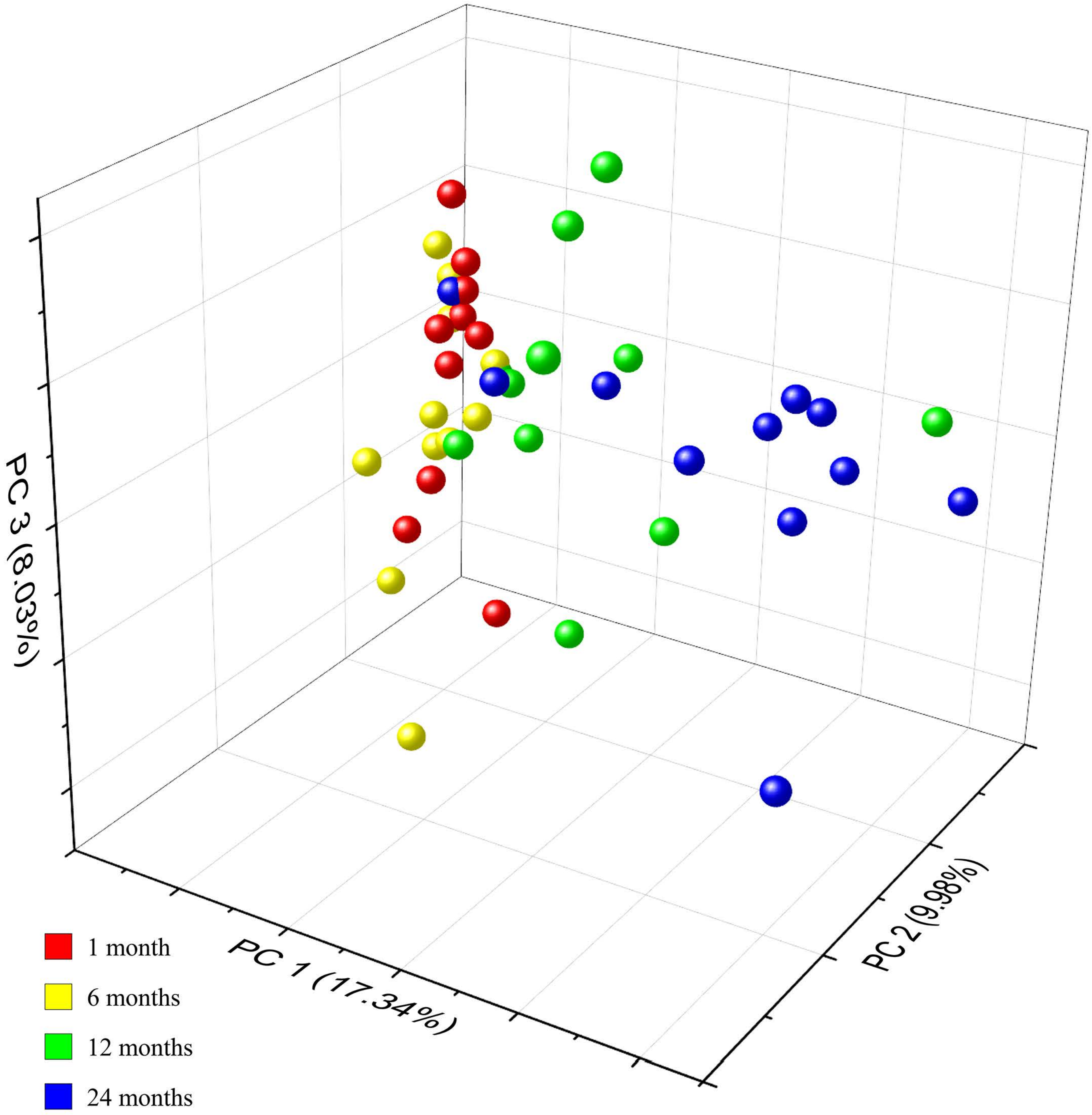

Figure S2. Beta-diversity of taxonomical diversity at the genus level of the 43 infant gut microbiomes. Bray-Curtis distances between samples are depicted through Principal Coordinate Analysis (PCoA), visualizing the progression of diversity from a few weeks of life (1 months) to post-weaning stage (12-24 months).

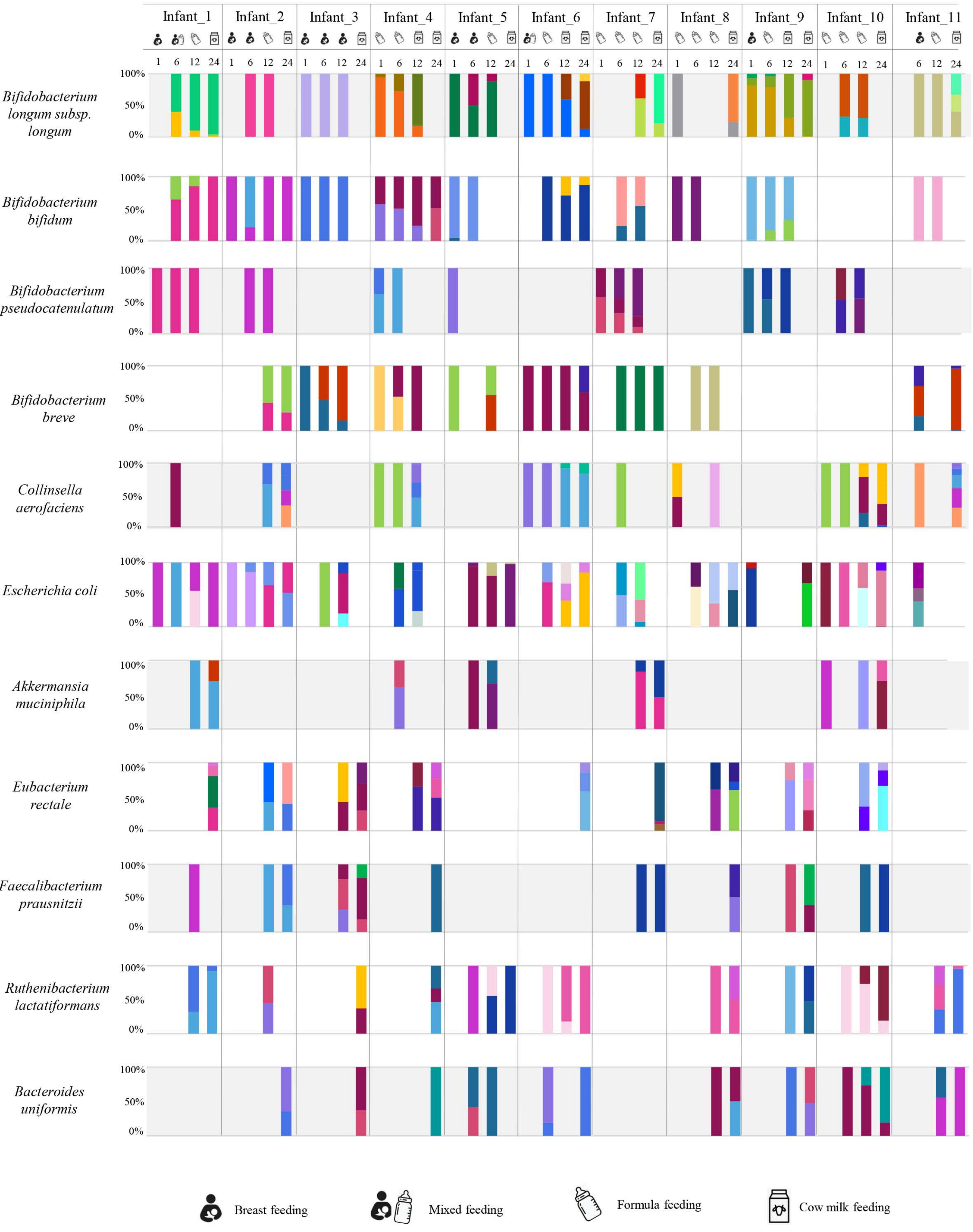

Figure S3. Strain-level composition and variability of the bacterial species identified with high prevalence in the infant's gut. Different strains (total of 203 reference strains, average of 18.5 per species) profiled in each of the 11 infants were highlighted with different colors. Infant's feeding type at each considered time-point of age (1-6-12-24-months) was reported at the top of the figure.

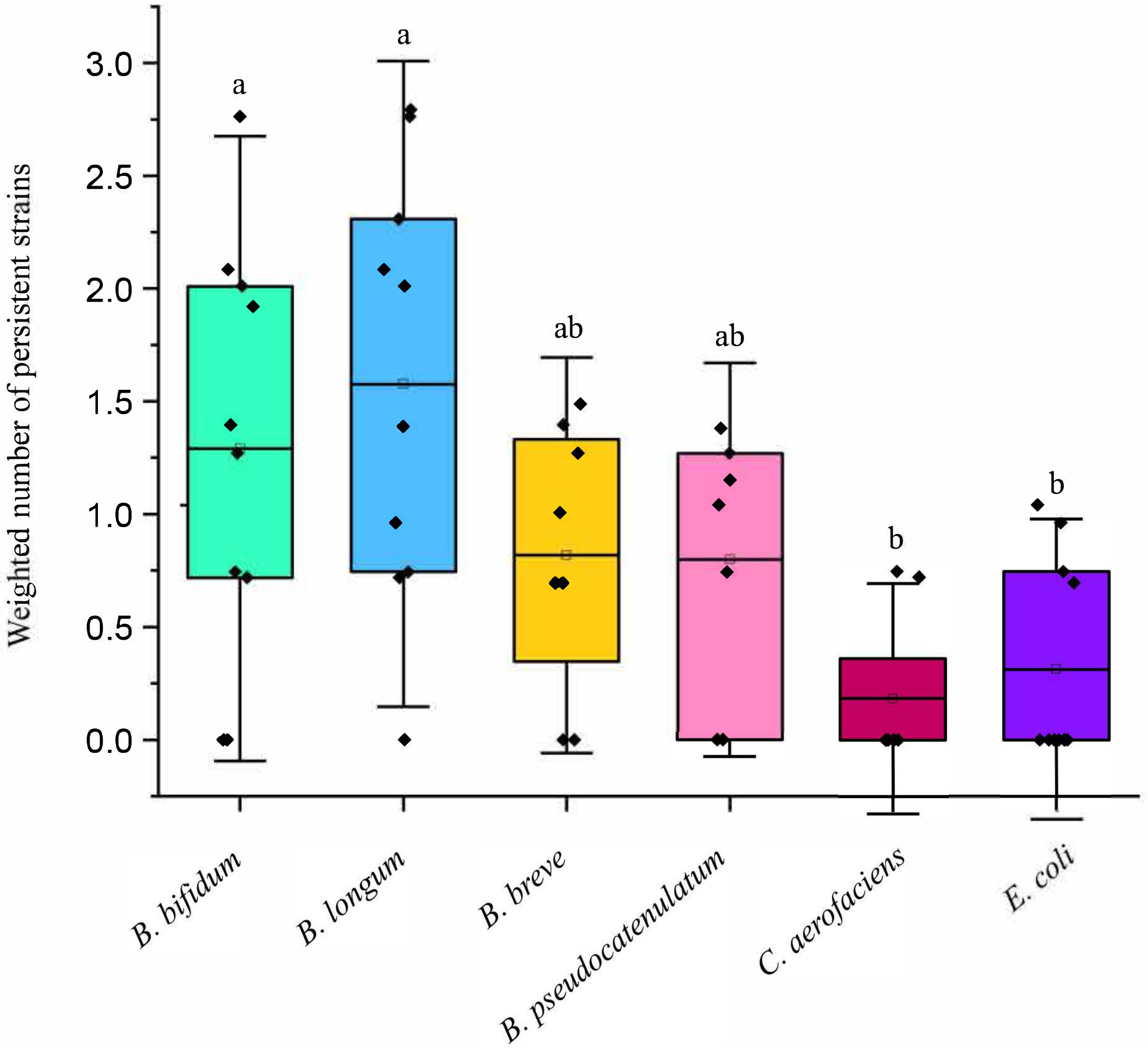

Figure S4. Difference in bacterial persistence between bifidobacterial and non-bifidobacterial strains throughout the infant weaning. After accounting for sequencing depth, the (weighted) number of strains shared between the pre- and post-weaning stages (n=11 for each sampling time) were compared among the six microbial species characterizing the infant gut microbiota (prevalence > 40 %). The boxes are determined by the 25th and 75th percentiles. The whiskers are determined by 1.5 interquartile range (IQR). The line in the boxes represents the median, while the square represents the average. Different lowercase letters indicate significant differences at p-value < 0.05 according to the Kruskal–Wallis with Dunn's post-hoc test and the Benjamini-Hochberg correction.

Figure S4

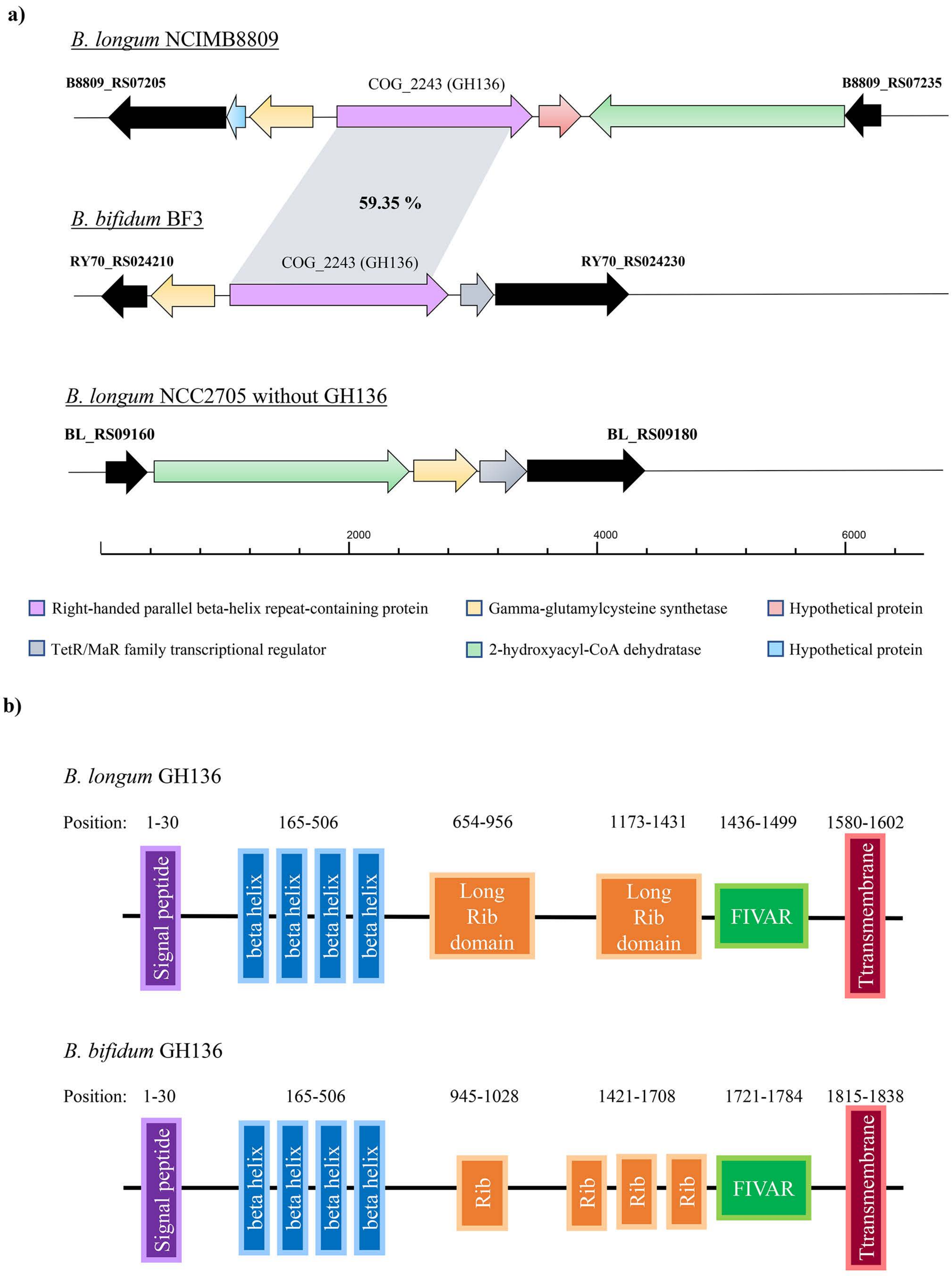

Figure S5. Locus map of *B. bifidum* and *B. longum* containing the GH136 and its main protein domains. Panel a shows the structure of the genomic region belonging to *B. longum* and *B. bifidum* chromosomes in which GH136 has been identified along with the genetic maps of a *B. longum* strain without GH136. The amino acid sequence identity percentage between GH136 retrieved from *B. longum* and *B. bifidum* are reported and genes are colored based on predicted function.

Panel b depicts a schematic illustration of the protein domain structure of the GH136 from *B. longum* and *B. bifidum*. Numbering refers to amino acid position.

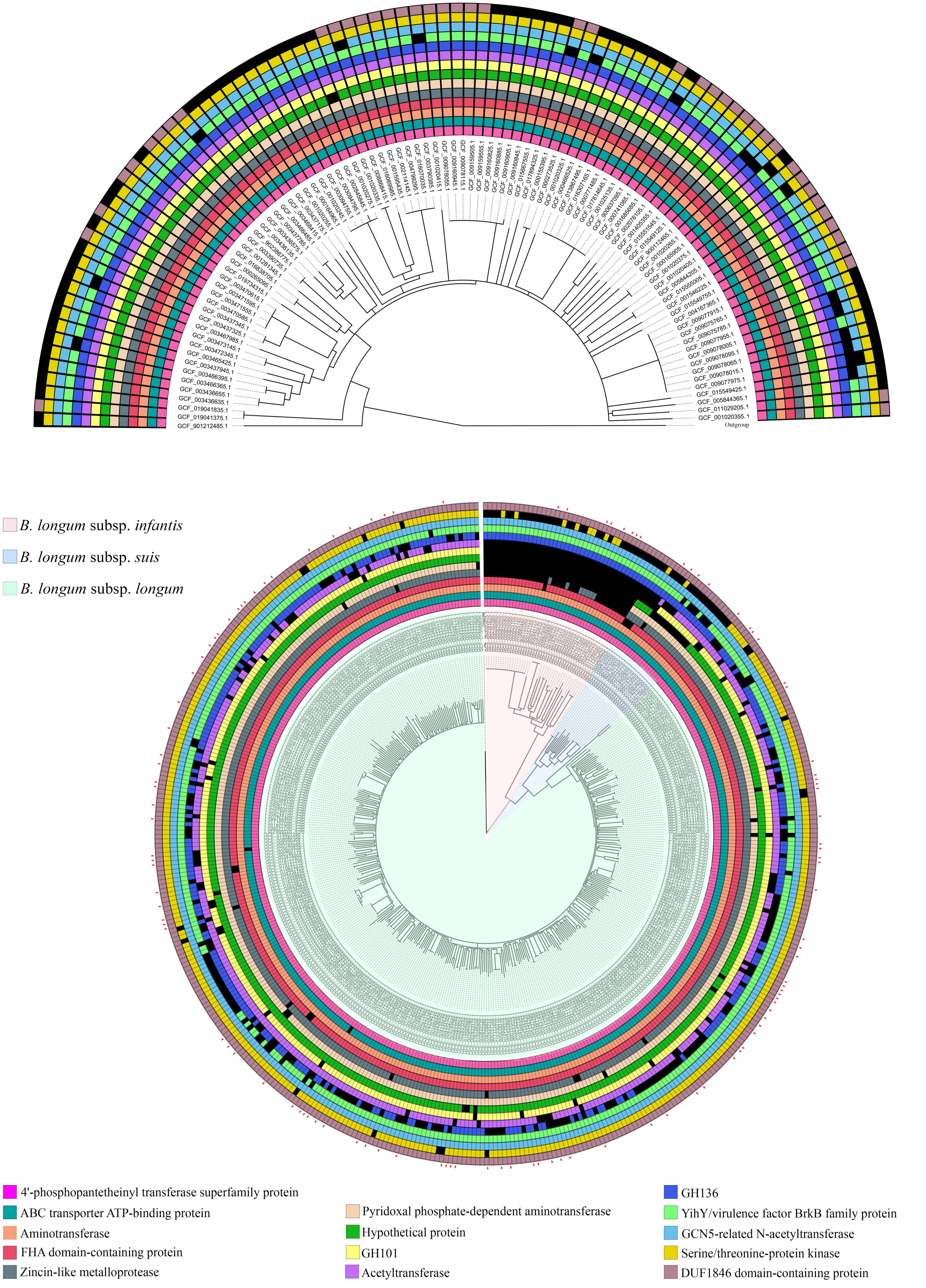

B. longum subsp. infantis
B. longum subsp. suis
B. longum subsp. longum
Outgroup
4'-phosphopantetheinyl transferase superfamily protein
ABC transporter ATP-binding protein
Aminotransferase
FHA domain-containing protein
Zincin-like metalloprotease
Pyridoxal phosphate-dependent aminotransferase
Hypothetical protein
GH101
Acetyltransferase
GH136
YihY/virulence factor BrkB family protein
GCN5-related N-acetyltransferase
Serine/threonine-protein kinase
DUF1846 domain-containing protein

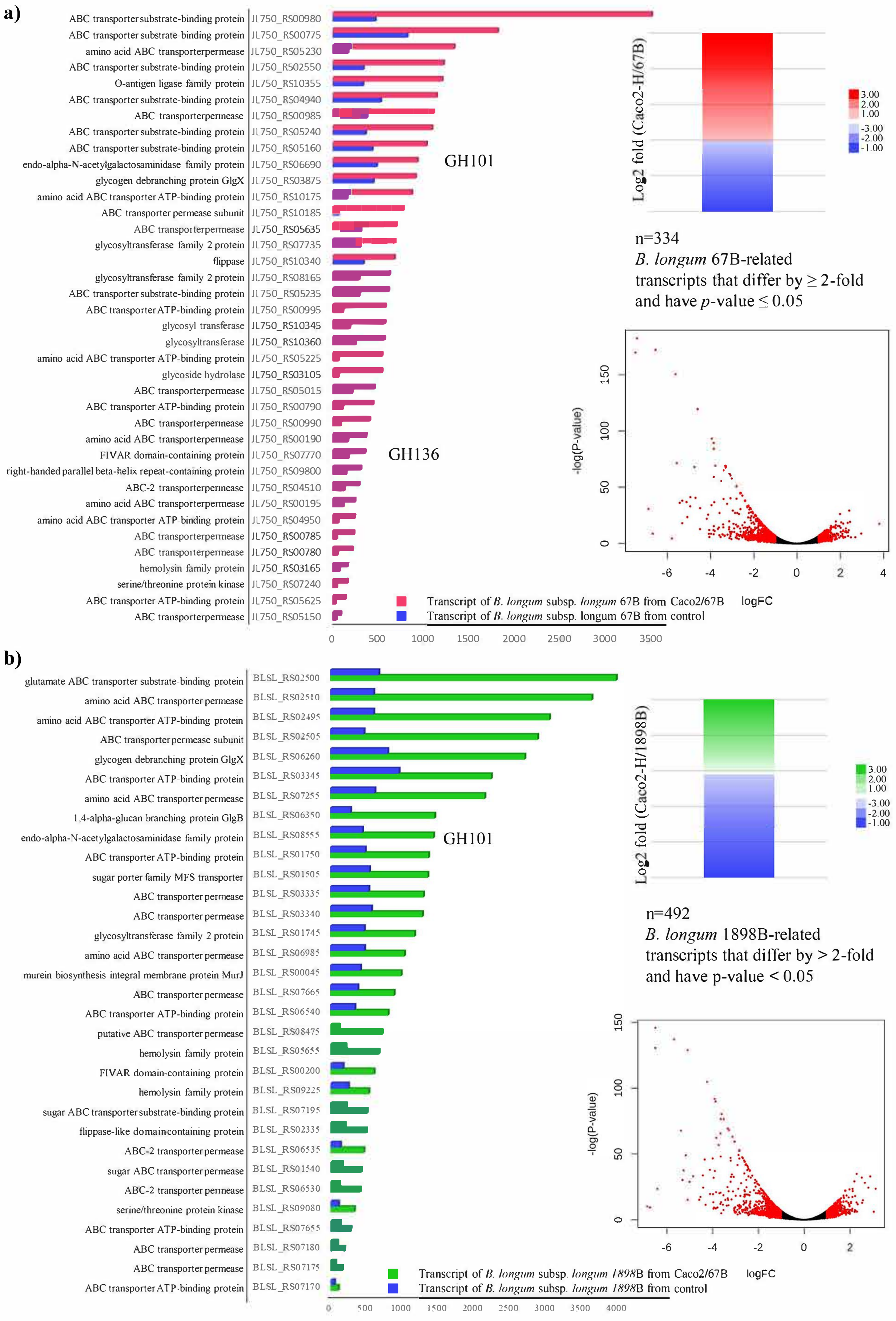

Figure S7. Differentially Expressed Genes (DEGs) in co-cultures of Caco2/HT29-MTX cell monolayers with *B. longum* subsp. *longum* PRL2022 and *B. longum* subsp. *longum* 1898B. Panel a and b report differential gene expression analyses of *B. longum* subsp. *longum* PRL2022 (panel a) and *B. longum* subsp. *longum* 1898B (panel b) transcriptomes upon the contact with Caco2/HT29-MTX cells, when compared with the respective control condition. After normalization of the row counts, genewise exact tests were compute to assess differential expression of each gene. Adjustement of p-values for multiple hypothesis was performed through the False Discovery Rate (FDR) procedure. Each Volcano plot depicts genes as points in two-dimensional space of statistical significance (vertical axis) vs. log2 fold change (horizontal axis). Significantly DEGs are highlighted in red color and are defined by cutoff of fold change > 2 (log2 FC > 1) and statistical significance (p-value) < 0.05. Up-regulated genes are to the right of x=0 and down-regulated gene are to the left of x=0.

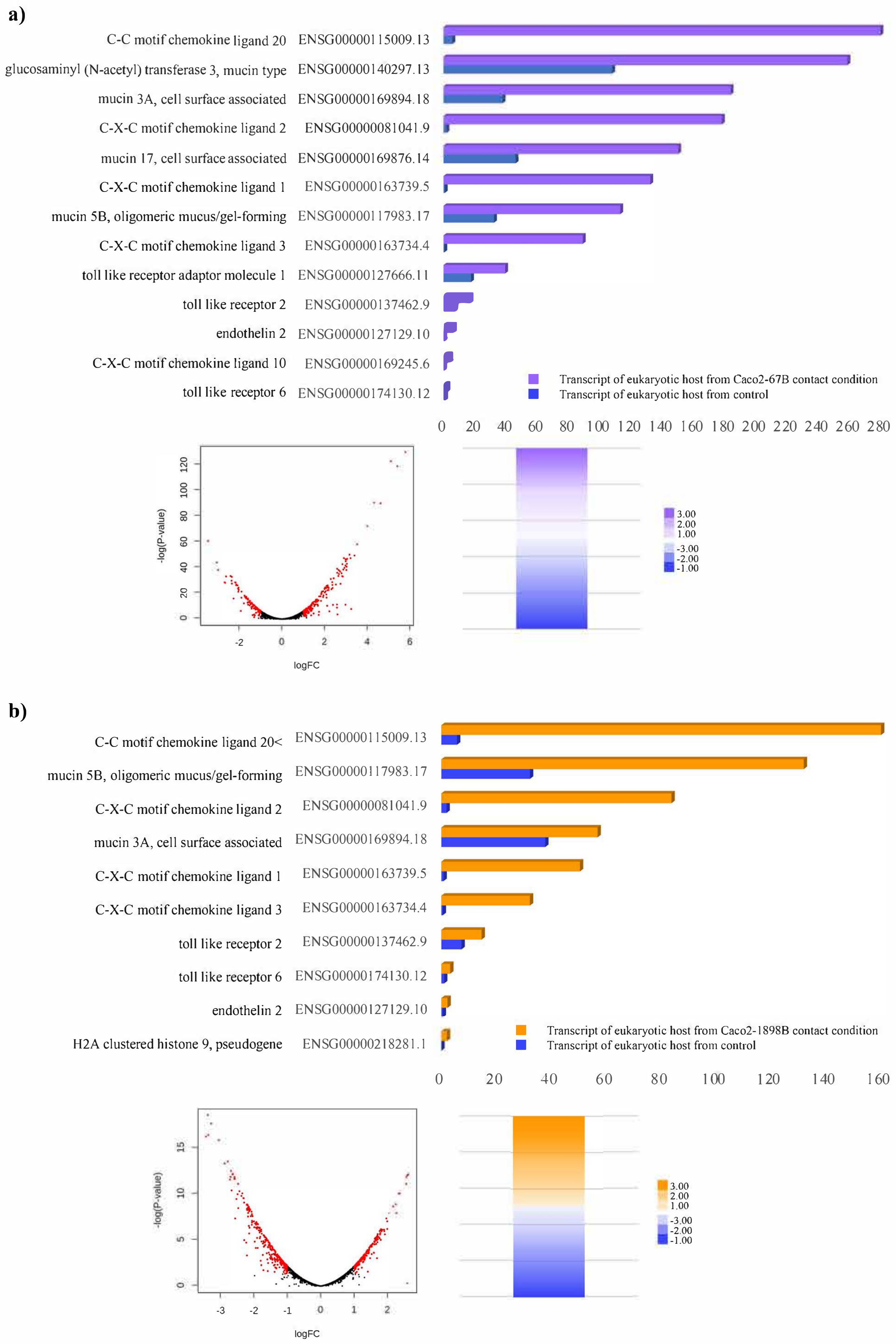

Figure S8. Differentially Expressed Genes (DEGs) from Caco2/HT29-MTX cell monolayers transcriptomes when placed in contact with *B. longum* subsp. *longum* PRL2022 and *B. longum* subsp. *longum* 1898B. Horizontal bar plot reports the main DEGs involved in mucin production and immune response from host human cell monolayers transcriptomes upon the contact with *B. longum* subsp. *longum* PRL2022 (panel a) and *B. longum* subsp. *longum* 1898B (panel b), when compared with control samples. After normalization of the row counts, genewise exact tests were compute to assess differential expression of each gene. Adjustement of p-values for multiple hypothesis was performed through the False Discovery Rate (FDR) procedure. Each Volcano plot depicts genes as points in two-dimensional space of statistical significance (vertical axis) vs. log2 fold change (horizontal axis). Significantly DEGs are highlighted in red color and are defined by cutoff of fold change > 2 (log2 FC > 1) statistical significance (p-value) < 0.05. Up-regulated genes are to the right of x=0 and down-regulated gene are to the left of x=0

References

1. Cabrera-Rubio R, Collado MC, Laitinen K, Salminen S, Isolauri E, Mira A. The human milk microbiome changes over lactation and is shaped by maternal weight and mode of delivery. American Journal of Clinical Nutrition. 2012 Sep 1;96(3):544–51.
2. Guo M, Miao M, Wang Y, Duan M, Yang F, Chen Y, et al. Developmental differences in the intestinal microbiota of Chinese 1-year-old infants and 4-year-old children. Scientific Reports. 2020 Dec 1;10(1).
3. Fallani M, Amarri S, Uusijarvi A, Adam R, Khanna S, Aguilera M, et al. Determinants of the human infant intestinal microbiota after the introduction of first complementary foods in infant samples from five European centres. Microbiology. 2011 May;157(5):1385–92.
4. Mancabelli L, Tarracchini C, Milani C, Lugli GA, Fontana F, Turroni F, et al. Multi-population cohort meta-analysis of human intestinal microbiota in early life reveals the existence of infant community state types (ICSTs). Computational and Structural Biotechnology Journal [Internet]. 2020 Jan 1 [cited 2021 Sep 20];18:2480–93. Available from: https://pubmed.ncbi.nlm.nih.gov/33005310/
5. Yang R, Gao R, Cui S, Zhong H, Zhang X, Chen Y, et al. Dynamic signatures of gut microbiota and influences of delivery and feeding modes during the first 6 months of life. Physiological genomics. 2019;51(8):368–78.
6. Stewart CJ, Ajami NJ, O'Brien JL, Hutchinson DS, Smith DP, Wong MC, et al. Temporal development of the gut microbiome in early childhood from the TEDDY study. Nature. 2018 Oct 25;562(7728):583–8.
7. Nilsen M, Saunders CM, Angell IL, Arntzen M, Lødrup Carlsen KC, Carlsen KH, et al. Butyrate Levels in the Transition from an Infant- to an Adult-Like Gut Microbiota Correlate with Bacterial Networks Associated with Eubacterium Rectale and Ruminococcus Gnavus. Genes. 2020 Nov 1;11(11):1–15.
8. Moore RE, Townsend SD. Temporal development of the infant gut microbiome. Open biology. 2019 Sep;9(9):190128.
9. Sangwan N, Xia F, Gilbert JA. Recovering complete and draft population genomes from metagenome datasets. Microbiome. 2016;4.
10. Milani C, Lugli GA, Fontana F, Mancabelli L, Alessandri G, Longhi G, et al. METAnnotatorX2: a Comprehensive Tool for Deep and Shallow Metagenomic Data Set Analyses. mSystems. 2021 Jun 29;6(3).
11. Lugli GA, Alessandri G, Milani C, Viappiani A, Fontana F, Tarracchini C, et al. Genetic insights into the dark matter of the mammalian gut microbiota through targeted genome reconstruction. Environmental microbiology. 2021 Jun 1;23(6):3294–305.
12. Shkoporov AN, Chaplin A V., Shcherbakova VA, Suzina NE, Kafarskaia LI, Bozhenko VK, et al. Ruthenibacterium lactatiformans gen. nov., sp. nov., an anaerobic, lactate-producing member of the family Ruminococcaceae isolated from human faeces. International journal of systematic and evolutionary microbiology. 2016 Aug 1;66(8):3041–9.
13. Tamburini S, Shen N, Wu HC, Clemente JC. The microbiome in early life: implications for health outcomes. Nature medicine. 2016 Jul 7;22(7):713–22.

14. Avershina E, Lundgård K, Sekelja M, Dotterud C, Storrø O, Øien T, et al. Transition from infant- to adult-like gut microbiota. Environmental microbiology. 2016 Jul 1;18(7):2226–36.

15. Nagarajan SN, Upadhyay S, Chawla Y, Khan S, Naz S, Subramanian J, et al. Protein kinase A (PknA) of Mycobacterium tuberculosis is independently activated and is critical for growth in vitro and survival of the pathogen in the host. The Journal of biological chemistry [Internet]. 2015 Apr 10 [cited 2021 Nov 11];290(15):9626–45. Available from: https://pubmed.ncbi.nlm.nih.gov/25713147/

16. Nezametdinova VZ, Zakharevich N v., Alekseeva MG, Averina O v., Mavletova DA, Danilenko VN. Identification and characterization of the serine/threonine protein kinases in Bifidobacterium. Archives of microbiology [Internet]. 2014 Feb [cited 2021 Nov 18];196(2):125–36. Available from: https://pubmed.ncbi.nlm.nih.gov/24395073/
