# Supplementary material for: Genetic strategies for sex-biased persistence of gut microbes across human life
Source: Nat Commun. 2023 Jul 14;14:4220. doi: 10.1038/s41467-023-39931-2 (PMC10349097; doi:10.1038/s41467-023-39931-2)
Supplement: Supplementary file 3 — Description of Additional Supplementary Files [file 41467_2023_39931_MOESM3_ESM.docx]

File Name: Supplementary Data 1
Description: Metagenomic samples list and metadata associated with the subjects considered in this study.

File Name: Supplementary Data 2
Description: Taxonomic classification of the 1-6 and 12-24 months-old gut microbiota of infants enrolled in this study.

File Name: Supplementary Data 3
Description: Metagenome-derived assembled genomes.

File Name: Supplementary Data 4
Description: List of the microbial strains used to build the 11 species-specific reference databases.

File Name: Supplementary Data 5
Description: The persistent pattern of bifidobacterial species across the infant weaning phase.

File Name: Supplementary Data 6
Description: Real-time based assay.

File Name: Supplementary Data 7
Description: Strain-level composition of the validation cohort. Infants in whom at least one strain persisted through the weaning phase are highlighted in bold. The association between persistence event and the infant's delivery mode was evaluated through Chi-Squared test reported below each table.

File Name: Supplementary Data 8
Description: Comparison between strain tracking analysis on infant dataset subsampled to 5,000,000 reads and the same dataset with all the available sequenced reads.

File Name: Supplementary Data 9
Description: Longitudinal strain-tracking analysis of the most relevant bifidobacterial species.

File Name: Supplementary Data 10
Description: Features of the 14 genes peculiar to gender-specific bifidobacterial species (*B. bifidum* and *B. longum* subsp. *longum*), and their prevalence in complete genomes and human populations.

File Name: Supplementary Data 11
Description: Transcriptomic data of *B. bifidum* growth on different carbon source.

File Name: Supplementary Data 12
Description: PERMANOVA analysis performed on the 12,415 cross-sectional metagenome data (reported in Table S1) to test the effect of population variables on the prevalence of *B. longum* subsp. *longum* and *B. bifidum*.

File Name: Supplementary Data 13
Description: Accounting for dairy food consumption and lactase persistence on *B. longum* subsp. *longum* and *B. bifidum* prevalence by using geographic regions as proxy variables.

File Name: Supplementary Data 14
Description: The involvement of the *B. longum*-derived GH136 gene in mother-to-infants vertical transmission events.

File Name: Supplementary Data 15
Description: Evaluation of reads number mapping specific *B. longum* genome sequences (*B. longum* subsp. *longum* AH1206 and AG1) in metagenomic fecal samples from publicly available clinical studies.

File Name: Supplementary Data 16
Description: Results of *B. longum* subsp. *longum* RNA sequencing and differentially expressed genes between co-cultures Caco2-HT29/B. longum subsp. longum 1898B, Caco2-HT29/*B. longum* subsp. *longum* PRL2022 and Control. After normalization of row counts, genewise exact tests were computed to assess the differential expression of each gene. P-values were adjusted for multiple hypotheses through the False Discovery Rate (FDR) procedure.

File Name: Supplementary Data 17
Description: Results of Human cell monolayers RNA sequencing and differentially expressed genes between co-cultures Caco2-HT29/*B. longum* subsp. *longum* 1898B, Caco2-HT29/*B. longum* subsp. *longum* PRL2022 and Control. After normalization of row counts, genewise exact tests were computed to assess the differential expression of each gene. P-values were adjusted for multiple hypotheses through the False Discovery Rate (FDR) procedure.
