# Supplementary material for: Genetic strategies for sex-biased persistence of gut microbes across human life
Source: Nat Commun. 2023 Jul 14;14:4220. doi: 10.1038/s41467-023-39931-2 (PMC10349097; doi:10.1038/s41467-023-39931-2)
Supplement: Supplementary file 21 — Reporting Summary [file 41467_2023_39931_MOESM21_ESM.pdf]

nature portfolio

Corresponding author(s): Marco Ventura

Last updated by author(s): Jun 7, 2023

Reporting Summary

Nature Portfolio wishes to improve the reproducibility of the work that we publish. This form provides structure for consistency and transparency in reporting. For further information on Nature Portfolio policies, see our Editorial Policies and the Editorial Policy Checklist.

Statistics

For all statistical analyses, confirm that the following items are present in the figure legend, table legend, main text, or Methods section.

n/a	Confirmed	
☐	☒	The exact sample size (*n*) for each experimental group/condition, given as a discrete number and unit of measurement
☐	☒	A statement on whether measurements were taken from distinct samples or whether the same sample was measured repeatedly
☐	☒	The statistical test(s) used AND whether they are one- or two-sided<br>*Only common tests should be described solely by name; describe more complex techniques in the Methods section.*
☐	☒	A description of all covariates tested
☐	☒	A description of any assumptions or corrections, such as tests of normality and adjustment for multiple comparisons
☐	☒	A full description of the statistical parameters including central tendency (e.g. means) or other basic estimates (e.g. regression coefficient) AND variation (e.g. standard deviation) or associated estimates of uncertainty (e.g. confidence intervals)
☒	☐	For null hypothesis testing, the test statistic (e.g. *F*, *t*, *r*) with confidence intervals, effect sizes, degrees of freedom and *P* value noted<br>*Give P values as exact values whenever suitable.*
☒	☐	For Bayesian analysis, information on the choice of priors and Markov chain Monte Carlo settings
☒	☐	For hierarchical and complex designs, identification of the appropriate level for tests and full reporting of outcomes
☒	☐	Estimates of effect sizes (e.g. Cohen's *d*, Pearson's *r*), indicating how they were calculated

Our web collection on statistics for biologists contains articles on many of the points above.

Software and code

Policy information about availability of computer code

Data collection	No algorithms or software were used.
Data analysis	Taxonomic profiling of shotgun reads, including those retrieved from publicly available shotgun datasets, was achieved with the METAnnotatorX2. Bacterial genome assembly was achieved through MEGAnnotator software and their quality was assess through checkM software (version 1.1.8). Dereplication of bacterial genome database was performed using DRep software (varsion 2.5.4). Strain Genome Explorer (StrainGE) toolkit (https://github.com/broadinstitute/strainge) and inStrain software (version 1.7.5) was used for the identification of bifidobacterial strains. Pangenome Analysis Pipeline (PGAP) v1.1 was employed for core-genome analysis. BWA (version 0.7.17-r1188) and Bowtie2 (version 2.4.0) was employed to mapping metagenomic reads against each reference sequence, and HTseq software (version 0.11.4) was used to compute reads row counts. Analysis of normalized reads count and false discovery rate correction was performed using DESeq2 (version 1.41.1). Human RnaSeq data (fastq) were aligned to the Human reference genome sequence (GRCh38.p13) using the splice-aware STAR algorithm (version 2.7.10a) and the quality of alignments was evaluated using Picard software tool (version 2.26.11). EdgeR package (version 3.6.1) was employed for raw counts normalization using TMM (Trimmed Mean of M-Values) and statistical-based differential gene expression analysis. The software SPSS version 25, and ORIGIN version 9.8.0.200 were used for statistical data analyses and graphing.

For manuscripts utilizing custom algorithms or software that are central to the research but not yet described in published literature, software must be made available to editors and reviewers. We strongly encourage code deposition in a community repository (e.g. GitHub). See the Nature Portfolio guidelines for submitting code & software for further information.

Data

Policy information about availability of data

All manuscripts must include a data availability statement. This statement should provide the following information, where applicable:

- Accession codes, unique identifiers, or web links for publicly available datasets
- A description of any restrictions on data availability
- For clinical datasets or third party data, please ensure that the statement adheres to our policy

Infant shotgun metagenomic sequencing and RNA-seq data of human intestinal cells, B. longum subsp. longum 1898 and PRL2022 are accessible through sequence read archive (SRA) accession code PRJNA833139 (https://www.ncbi.nlm.nih.gov/bioproject/PRJNA833139). GRCh38.p13 is available from https://www.ensembl.org/Homo_sapiens/Info/Index?db=core. B. longum subsp. longum 1898B and PRL2022 are accessible through https://www.ncbi.nlm.nih.gov/assembly/GCA_002075875.1 and https://www.ncbi.nlm.nih.gov/assembly/GCA_016759745.1, respectively. Carbohydrate-active enzyme (CAZy) database is available from http://www.cazy.org/.

Human research participants

Policy information about studies involving human research participants and Sex and Gender in Research.

Reporting on sex and gender	This study revealed significant sex-based differences in intestinal persistence of specific microbial phylotypes. Information regarding sex of participants was recovered from the data reported in previously published studies or from infant parents, and disaggregated sex data were available in the source data.
Population characteristics	Longitudinal metagenomic fecal samples (43 analyzed in this study) were collected from 384 infants aged 0-2 years, including 188 females and 196 males. All infants were regarded as healthy, delivered vaginally at term, and were not subjected to antibiotic treatment. In addition, a total of 12,415 cross-sectional fecal samples (6,545 from female and 5,870 from males) derived from infants (0-4 years old, 1,456 female and 1,541 males), children (5-18 years old, 434 females and 484 males), adults (19-55 years old, 3,379 females and 2,768 males), and elderly (56-90 years old, 1,276 females and 1,077 males) were selected from publicly available repositories. Specifically, only individuals defined as healthy (control) in their respective datasets were retained. In order to assess the effects of possible dairy food consumption and lactase persistence we used geographical region as a proxy parameter. Specifically, subsets of metagenomic samples from Southern Europe (n=417, 217 males and 200 females) and Northern Europe (n=413, 186 males and 227 females) were selected to account for lactase persistence, while fecal samples of subjects from China (n=831, 413 males and 418 females) were compared with those from Europe (n=830, 403 males and 427 females) to account for dairy consumption.<br>For public metagenomic datasets, all relevant population characteristics (e.g., age and sex) were retrieved from the publicly available associated study. For the 11 infants whose fecal samples were analyzed in the context of this study, information about sex, age, feeding type, delivery mode, and time of weaning were collected directly from parents.
Recruitment	A total of 11 healthy infants born after an uncomplicated pregnancy were recruited at the Central University Hospital of Asturias (Northern Spain) in the first medical consultation, which takes place in the first 10–15 days of the infant's life. Participants were enrolled on the basis of meeting inclusion criteria, which included vaginal delivery and full-term birth (37-40 gestational weeks). Exclusion criteria included: prescription of a therapeutic diet, assumption of antibiotics and pre- or probiotics during the sampling points considered in the study, incomplete dietary questionnaire.<br>Informed written consent was obtained from each infant parents before enrollment. Fecal samples were then collected at scheduled appointments from one month to two years after birth.
Ethics oversight	The study was approved by the Regional Ethical Committee of Asturias Public Health Service (Ref.Nº 51/18) and the Ethical Committee of CSIC (Ref 136/2018).

Note that full information on the approval of the study protocol must also be provided in the manuscript.

Field-specific reporting

Please select the one below that is the best fit for your research. If you are not sure, read the appropriate sections before making your selection.

☒ Life sciences ☐ Behavioural & social sciences ☐ Ecological, evolutionary & environmental sciences

For a reference copy of the document with all sections, see nature.com/documents/nr-reporting-summary-flat.pdf

Life sciences study design

All studies must disclose on these points even when the disclosure is negative.

Sample size	This study investigated 1,149 longitudinal fecal metagenomes from 384 healthy infants (186 females and 196 males) to inspect the gut microbiome compositional changes at species level during the first two years of life. Additionally, 12,415 cross-sectional metagenomic fecal samples from roughly 3,000 infants (0-4 years old), 918 children (5-18 years old), 6,147 adults (19-55 years old), and 2,353 elderly (56-90 years old) (52 % females and 48 % males) were employed to investigate sex-related persistence of intestinal microbial taxa across human

	population at different stages of host life. Moreover, to validate the link between specific microbial genetic features and sex-associated intestinal persistence, we analyzed a total of 35 publicly available metagenomic fecal samples from healthy humans (48% females) who received daily oral doses of viable bacterial cells. As this study involves a large number of publicly available data, the sample size was mainly determined by the availability of resources or based on prior experience with similar study. Therefore, no statistical methods were used to predetermine sample size.
Data exclusions	In order to guarantee high resolution and consistency of the input data, we selected shotgun metagenomics data sets only based on the Illumina sequencing platform. Moreover, samples from individuals with reported intestinal morbidity were excluded a priori. Following analyses, metagenomic samples with less than 5,000,000 sequenced reads were excluded.
Replication	All analyses were successfully replicated at least twice. In vitro experiments evaluating bacterial growth on different carbon sources and following host cells contact were conducted in triplicate. All data required to reproduce our analyses are publicly available without restrictions.
Randomization	Randomization was not applicable, as this study was an observational study based on a stratified random sampling of human population aged (0-90 years) investigating the association between bacterial intestinal persistence and host sex. Therefore, no experimental groups were used.
Blinding	Blinding was not applicable, as the study had no experimental group allocation.

Reporting for specific materials, systems and methods

We require information from authors about some types of materials, experimental systems and methods used in many studies. Here, indicate whether each material, system or method listed is relevant to your study. If you are not sure if a list item applies to your research, read the appropriate section before selecting a response.

Materials & experimental systems

n/a	Involved in the study
☒	☐ Antibodies
☐	☒ Eukaryotic cell lines
☒	☐ Palaeontology and archaeology
☒	☐ Animals and other organisms
☒	☐ Clinical data
☒	☐ Dual use research of concern

Methods

n/a	Involved in the study
☒	☐ ChIP-seq
☒	☐ Flow cytometry
☒	☐ MRI-based neuroimaging

Eukaryotic cell lines

Policy information about cell lines and Sex and Gender in Research

Cell line source(s)	Human cell lines were purchased from ATCC. Caco-2 are epithelial cells isolated from colon tissue derived from a 72-years old, White, male with colorectal adenocarcinoma, while HT29-MTX are mucin-secreting goblet cell line derived from a 49-years old, White, female with colon carcinoma.
Authentication	Short tandem repeat (SRT) profiling was performed as part of ATCC routine authentication procedures
Mycoplasma contamination	All cell lines tested negative for mycoplasma contamination
Commonly misidentified lines (See ICLAC register)	None misidentified cell lines were used
